# Supplementary material for: Proteomic analysis of cardiometabolic biomarkers and predictive modeling of severe outcomes in patients hospitalized with COVID-19
Source: Res Sq. 2022 Jun 1:rs.3.rs-1657002. Preprint. [Version 1] doi: 10.21203/rs.3.rs-1657002/v1 (PMC9176658; doi:10.21203/rs.3.rs-1657002/v1)
Supplement: Supplement 1 [file NIHPPRS1657002v1-supplement-1.pdf]

Supplementary Files

This is a list of supplementary files associated with this preprint. Click to download.

- Supplement.pdf
